# Ethylene is Involved in Brassinosteroids Induced Alternative Respiratory Pathway in Cucumber (*Cucumis sativus* L.) Seedlings Response to Abiotic Stress

**DOI:** 10.3389/fpls.2015.00982

**Published:** 2015-11-10

**Authors:** Li-Jie Wei, Xing-Guang Deng, Tong Zhu, Ting Zheng, Peng-Xu Li, Jun-Qiang Wu, Da-Wei Zhang, Hong-Hui Lin

**Affiliations:** State Key Laboratory of Hydraulics and Mountain River Engineering, Key Laboratory for Bio-Resource and Eco-Environment, College of Life Science, Ministry of Education, Sichuan UniversityChengdu, China

**Keywords:** alternative oxidase, environmental stress, cucumber, brassinosteroid, ethylene

## Abstract

Effects of brassinosteroids (BRs) on cucumber (*Cucumis sativus* L.) abiotic stresses resistance to salt, polyethylene glycol (PEG), cold and the potential mechanisms were investigated in this work. Previous reports have indicated that BRs can induce ethylene production and enhance alternative oxidase (AOX) pathway. The mechanisms whether ethylene is involved as a signal molecule which connected BR with AOX in regulating stress tolerance are still unknown. Here, we found that pretreatment with 1 μM brassinolide (BL, the most active BRs) relieved stress-caused oxidative damage in cucumber seedlings and clearly enhanced the capacity of AOX and the ethylene biosynthesis. Furthermore, transcription level of ethylene signaling biosynthesis genes including ripening-related *ACC* synthase1 (*C*_*S*_*ACS1*), ripening-related *ACC* synthase2 (*C*_*S*_*ACS2*), ripening-related *ACC* synthase3 (*C*_*S*_*ACS3*), 1-aminocyclopropane-1-carboxylate oxidase1 (*C*_*S*_*ACO1*), 1-aminocyclopropane-1-carboxylate oxidase2 (*C*_*S*_*ACO2*), and *C*_*S*_*AOX* were increased after BL treatment. Importantly, the application of the salicylhydroxamic acid (SHAM, AOX inhibitor) and ethylene biosynthesis inhibitor aminooxyacetic acid (AOA) decreased plant resistance to environmental stress by blocking BRs-induced alternative respiration. Taken together, our results demonstrated that ethylene was involved in BRs-induced AOX activity which played important roles in abiotic stresses tolerance in cucumber seedlings.

## Introduction

In both natural and agricultural environments, plants are challenged by heat, cold, drought, and high salinity throughout their life cycles, influencing plant growth and productivity (Xia et al., 2009). To survive from environmental stresses, plants have evolved biochemical and physiological mechanisms to activate tolerance against environmental stresses (Boyer, 1982). It is well known that stress conditions along with reactive oxygen species (ROS) generation, such as superoxide anion (O${}_{2}^{\cdot -}$), hydrogen peroxide (H_2_O_2_) and other free radicals are harmful for plant organisms and making severely damage (Scandalios, 1993; Zhang et al., 2001). In order to eliminate the ROS damage, plants have evolved the antioxidant enzymes that are responsible for scavenging superfluous ROS accumulation under environmental stresses, including guaiacol peroxidase (GPX), catalase (CAT), superoxide dismutase (SOD), ascorbate peroxidase (APX), and peroxidase (POD) (Zhang et al., 2015). However, salicylic acid (SA) and abscisic acid (ABA) treatments can lead to a temporary elevation of H_2_O_2_, resulting in an enhanced tolerance to heat, oxidative stress, high light and salt, which suggested that ROS may function as a second messenger in the signaling pathways of plant stress responses (Dat et al., 1998; Apel and Hirt, 2004).

Ethylene is a gaseous phytohormone that is synthesized in almost all plant organs. It not only plays a role in plant growth and development, but also is involved in plant responses to biotic and abiotic stresses such as salt, chilling and ozone (Kendrick and Chang, 2008). Methionine is the starting point in the ethylene biosynthetic pathway, which is converted into S-adenosylmethionine (SAM) by methionine adenosyltransferases. S-adenosylmethione was changed to 1-amioncyclopropane-1-carboxylic acid (ACC) by ACC synthase (ACS) and then to ethylene by ACC oxidase (ACO) (Lin et al., 2010; Wang et al., 2012). Some studies have showed that ethylene induced the cyanide-resistant respiration in ripening fruits (Solomos and Laties, 1976). Several studies have also proposed that the increased ethylene production during fruit ripening might lead to increased HCN level, which in turn inhibited cytochrome oxidase and induced the activity of alternative oxidase (Mizutani et al., 1987; Pirrung and Brauman, 1987; Yip and Yang, 1988). It also has been showed that ethylene is closely connected with AOX induction under stress treatments, including salinity, chilling, pathogen infection, and ozone (Ederli et al., 2006; Wang et al., 2012). Recent studies reported that ethylene accumulation could be induced by BRs treatment in tomato fruit (Zhu et al., 2015).

Respiration plays a pivotal role in driving the cellular metabolism and transport processes. The electron transport system of the plant inner mitochondrial membrane contains two terminal oxidases, including cytochrome oxidase (COX) and alternative oxidase (AOX) (Clifton et al., 2006). It has been confirmed that AOX is the terminal oxidase in the alternative pathway (AP). AP is a non-phosphorylating electron transport chain (ETC) that diverts at the ubiquinone pool and uses AOX (McIntosh, 1994; Wang et al., 2012). As we know that the AP plays an important role in fruit ripening, plant thermogenesis and responses to stress conditions, including salinity, drought, low temperature, ozone, and pathogen invasion (Yoshida et al., 2007). Previous researches have investigated that AOX was involved in optimizing photosynthesis and it might has a pivotal role in relieving the over-reduction of chloroplasts (Yoshida et al., 2008; Giraud et al., 2009; Zhang et al., 2010). In addition, it indicated that AOX could also scavenge ROS to enhance the plant stress tolerance (Xu et al., 2012b).

Plant steroid hormones, brassinosteroids (BRs) were involved in a broad spectrum of plant growth and development. In addition, it played an important role in a variety of plant physiological processes and adaptation to various abiotic and biotic stresses (Zhang et al., 2015). While, most studies on the role of BRs in stress responses depend on exogenous addition of BRs or their biosynthesis inhibitor Recently, some studies found that BRs induce a transient increase in H_2_O_2_ content. It has been suggested that BRs-induced ROS generation is important for BRs-induced stress tolerance in cucumber, tobacco and tomato (Nakashita et al., 2003; Zhou et al., 2014; Deng et al., 2015). So we hypothesized that there might be some connections among ethylene, ROS, AOX, and BR signaling during the induction of plant stress tolerance. Here, this assumption was examined and our experimental results demonstrated that ethylene might be involved in BRs-induced alternative pathway under environmental stress. We also testified the possible regulation and physiological function of the alternative pathway in BRs-induced stress tolerance.

## Materials and methods

### Plant material and growth conditions

Plants growth as described (Xu et al., 2012b). Seeds of cucumber (*Cucumis sativus* L. cv. Jihong no. 2) were surface-sterilized for 15 min in 1% (w/v) NaClO, and then germinated on water-moistened filter paper. The plants were grown in a growth chamber at a 16-h photoperiod (100 μM m^−2^ s^−1^), temperature of 25°C/17°C (day/night).

Cucumber seedlings were cultivated with half-strength Hoagland's nutrient solution until the three-leaf stage. Then, the cucumber plants were treated with Brassinolide (BL, the most active BRs; Chuo-Ku, Osaka, Japan) with 0.1, 0.5, 1, 5, or 10 μM solutions on leaves, while the control seedlings were sprayed with distilled water. Twelve hours after spraying, all the seedlings were exposed to salt (200 mM NaCl), PEG (16% PEG 6000), and cold stresses (at 4°C in a controlled growth chamber with a relative humidity of 70%) for 3 d. The third leaf of cucumber seedlings was used for the following experiments.

### Treatment

Salicylhydroxamic acid (SHAM, an inhibitor of the AOX pathway), dimethylthiourea (DMTU, an H_2_O_2_ scavenger), DPI (an NADPH oxidase inhibitor), aminooxyacetic acid (AOA, an ethylene biosynthesis inhibitor), and ethylene were purchased from Sigma (StLouis, USA). 1 mM SHAM inhibits the AOX activity, as this concentration is sufficiently low to avoid the possible side effects (Møller et al., 1988). For BL+SHAM treatment, seedlings were pretreated with 1 mM SHAM, 24 h later were treated with 1 μM BL for another 12 h. Then, these plants were exposed to stress conditions as described earlier. To investigate the role of ROS in the resistance, leaves were pretreated with 100 μM DPI or 5 mM DMTU, then treated with 1 μM BL; 12 h later plants were treated with 10 mM H_2_O_2_. Then 1 mM AOA was sprayed to the seedlings for 12 h at room temperature. For the ethylene treatment, one lot seedling was incubated in 3.5 μl l^−1^ ethylene solution in a closed container at room temperature for 12 h. Then, these plants were exposed to stress conditions as described earlier.

### Cucumber leaf respiration measurements

Respiratory oxygen consumption was measured using Clark-type electrodes (Hansatech, King's Lynn, UK) as previously described (Xu et al., 2012b). Approximately 30 mg of leaves were cut into small pieces, then pretreated with 5 mL deionized water for 15 min in order to eliminate wound-induced respiration. Measurements were done at 25°C in a final volume of 1.5 mL phosphate buffer (pH 6.8) and the cuvette was tightly closed to prevent diffusion of oxygen from the air. Inhibitors of the cytochrome pathway (1 mM KCN) and the alternative pathway (0.5 mM n-propyl gallate, nPG) were used. The total respiration (Vt) is defined as O_2_ uptake rate by cucumber leaves without any inhibitor. Next, 1 mM KCN was added to obtain the O_2_ uptake rate defined as V_0_. Residual respiration (Vres) was defined as O_2_ uptake in the presence of both 1 mM KCN and 0.5 mM nPG. The capacity of the cytochrome pathway (Vcyt) and the alternative pathway (Valt) were calculated by the formula: Vcyt = Vt–V_0_; Valt = V_0_–Vres. The Vres in our experiment was always low, and was negligible relative to other respirations. Therefore, the Vres was not shown.

### Determination of ethylene emission

Ethylene was measured as previously described (Xu et al., 2012b). For ethylene production, intact seedlings were placed in 100 mL closed container and incubated at room temperature (25°C) for 1 h. Then, a 1 mL sample of gas from each container headspace was injected into a FID gas chromatograph (Agilent 6890 Series GC System, Salem, MA) equipped with an activated alumina SS column. The carrier gas (helium) flow rate was 0.5 mL s^−1^. The detector and injector were operated at 100°C, and the oven was at 50°C.

### Analysis of chlorophyll fluorescence

Chlorophyll fluorescence was determined with an imaging pulse amplitude modulated fluorometer (IMAG-MINI; Heinz Walz, Germany). For measurement of *F*_*v*_/*F*_*m*_, plants were dark adapted for 30 min. Minimal fluorescence (*F*_*o*_) was measured during the weak measuring pulses, and maximal fluorescence (*F*_*m*_) was measured by a 0.8 s pulse of light at about 4000 μmol m^−2^ s^−1^. An actinic light source was then applied to obtain steady-state fluorescence yield (*F*_*s*_), after which a second saturation pulse was applied for 0.7 s to obtain light adapted maximum fluorescence ($F_{m}^{\prime }$). *F*_*v*_/*F*_*m*_ and non-photochemical quenching (NPQ) were calculated as *F*_*m*_– *F*_*o*_/*F*_*m*_ and (*F*_*m*_/$F_{m}^{\prime }$) – 1, respectively (Fu et al., 2014).

### RNA extraction and quantitative real-time PCR (qRT–PCR)

Total RNA was extracted from cucumber leaves according to a previously described method (Zhang et al., 2010). RNA contents were calculated by measuring the absorbance value taken at 260 nm. All gene expression levels were measured by qRT-PCR. The Ct (threshold cycle), defined as the PCR cycle at which a statistically significant increase of reporter fluorescence was first detected, was used as a measure for the starting copy numbers of the target gene. Relative quantitation of gene expression was performed using the comparative Ct method. Three biological replicates were performed for each experiment. β-actin was used as internal controls. All the qRT-PCR primers are listed in Supplemental Table S1.

### Histochemical staining of superoxide and H_2_O_2_

Superoxide and H_2_O_2_ levels were visually detected with nitro blue tetrazolium (NBT) and 3, 3-diaminobenzidine (DAB), respectively, as previously described (Yang et al., 2004). cucumber leaves were excised at the base with a razor blade and vacuum infiltrated with NBT (0.5 mg mL^−1^) solution for 2 h, or DAB (2 mg mL^−1^) solution for 8 h. Leaves were then decolorized in boiling ethanol (95%) for 20 min.

### Oxidative damage estimation

The H_2_O_2_ content of leaves was measured as described (Xu et al., 2012b). Approximately 0.5 g of fresh cucumber leaves was cut into small pieces and homogenized in an ice bath with 5 mL 0.1% (w/v) trichloroacetic acid (TCA). The homogenate was centrifuged at 12,000 g for 20 min at 4°C. In addition, 0.5 mL of the supernatant was added to 0.5 mL 10 mM potassium phosphate buffer (pH 7.0) and 1 mL 1 M KI. The absorbance of the supernatant was read at 390 nm.

Electrolyte leakage (EL) was tested as previously described (Cao et al., 2009). After measuring the conductivity of the leaves, the leaves were boiled for 15 min to achieve 100% electrolyte leakage.

Lipid peroxidation was estimated by measuring the malondialdehyde (MDA) level as described previously (Velikova et al., 2000).

Cell death was showed by trypan blue staining. Leaves submerged in trypan blue (1.25 mg mL^−1^; Sigma) were heated in a boiling water bath for 2 min. The samples were then decolorized in boiling ethanol (95%) for 15 min.

### Antioxidant enzyme extraction and activity assay

For the enzyme assays, 0.3 g of leaf were ground with 3 mL ice-cold 25 mM.

HEPES buffer (pH 7.8) containing 0.2 mM EDTA, 2 mM AsA, and 2% PVP. The homogenates were centrifuged at 4°C for 20 min at 12,000 g, and the resulting supernatants were used for the determination of enzymatic activity (Xia et al., 2009). Superoxide dismutase (SOD), ascorbate peroxidase (APX), catalase (CAT), guaiacol peroxidase (GPX), and peroxidase (POD) activities were assayed as described previously (Wang et al., 2011).

### Statistical analysis

Means of three triplicates were measured. Student's *t*-test was used for comparison between different treatments. A difference was considered to be statistically significant when *P* < 0.05 or very significant when *P* < 0.01.

## Results

### Suitable concentration of BRs pretreatment improves plant abiotic stresses tolerance

**Figure 1:**
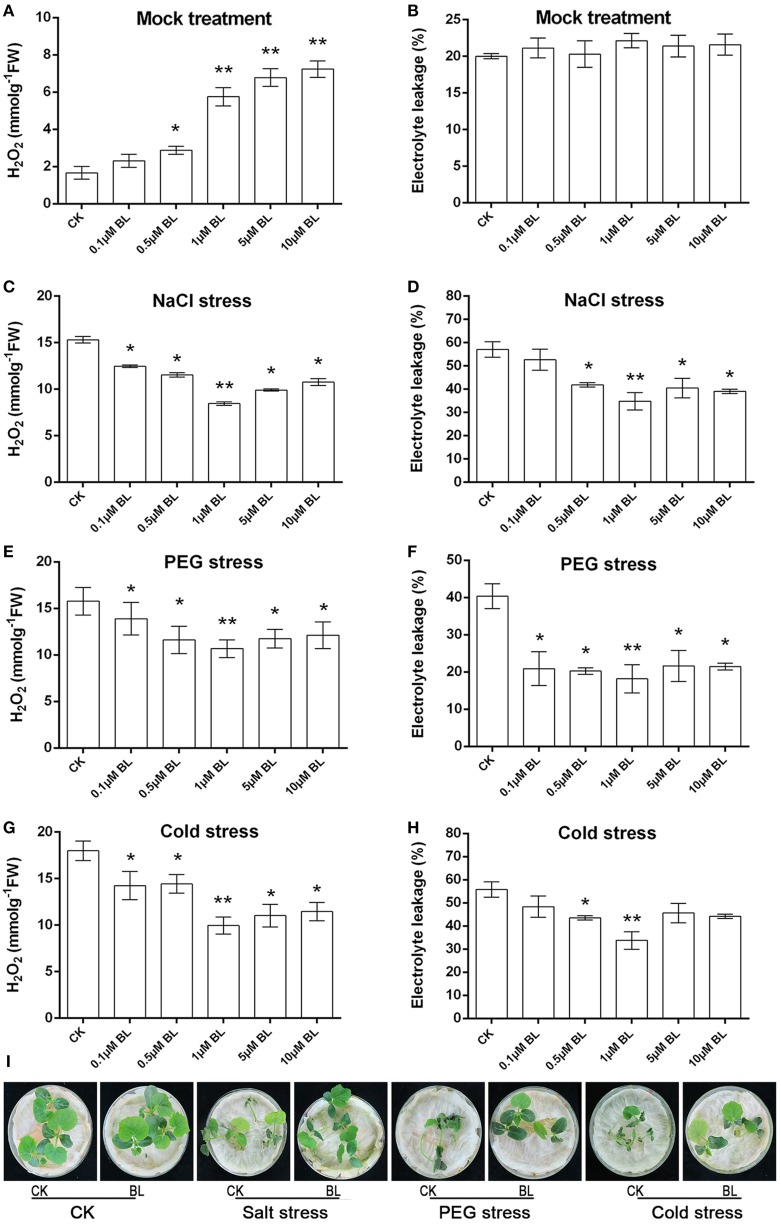
**At the three-leaf stage, cucumber seedlings were sprayed with 0.1, 0.5, 1, 5, or 10 μM BL solutions on leaves, while the control plants were sprayed with distilled water**. 12 h later, the seedlings were challenged with salt (200 mM NaCl), PEG (16% PEG6000), or cold (4°C) stresses for 3d, the third leaf was used for H_2_O_2_ content **(A,C,E,G)**, electrolyte leakage (EL) **(B,D,F,H)**. **(I)** Representative phenotypes of cucumber seedlings with or without stresses treatments. Data are the mean ± SD of three biological repeats; the significant difference was analyzed by Student's *t*-test (^*^P < 0.05, ^**^P < 0.01) and compared to control (CK).

H_2_O_2_ content and electrolyte leakage were detected following pretreatment with water, different concentrations of BL for 12 h. As shown in Figure 1A, under normal growth condition, H_2_O_2_ content increased significantly with increasing concentrations of BL pretreatment as compared with control, while there was no significant difference in electrolyte leakage (Figure 1B). In order to study the roles of BRs in plant stress tolerance, cucumber seedlings were exposed to different stress conditions, including salt (200 mM NaCl), PEG (16% PEG6000), and cold stresses (4°C), after being pretreated with different concentrations of BL for 12 h. Then 3 days later, we detected H_2_O_2_ content and electrolyte leakage. As shown in Figures 1C–H, under abiotic stresses the content of H_2_O_2_ and electrolyte leakage were repressed when seedlings were with 0.1, 0.5, 1, 5, or 10 μM BL treatment, especially under 1 μM BL treatment. In addition, pretreatment with 5 or 10 μM BL did not alleviate stress-induced injury (Supplemental Figure S1A). Cucumber seedlings pretreatment with 1 μM BL increased H_2_O_2_ content in the early stage, 72 h abiotic stresses treatment later, the level of H_2_O_2_ was lower than the control (Supplemental Figure S1B). The findings showed that seedlings pretreated with 1 μM BL displayed stronger tolerance to the three types of abiotic stresses (Figure 1I), and then the concentration of 1 μM BL was used in the following research.

### Effects of BL on the AOX capacity and ethylene production

**Figure 2:**
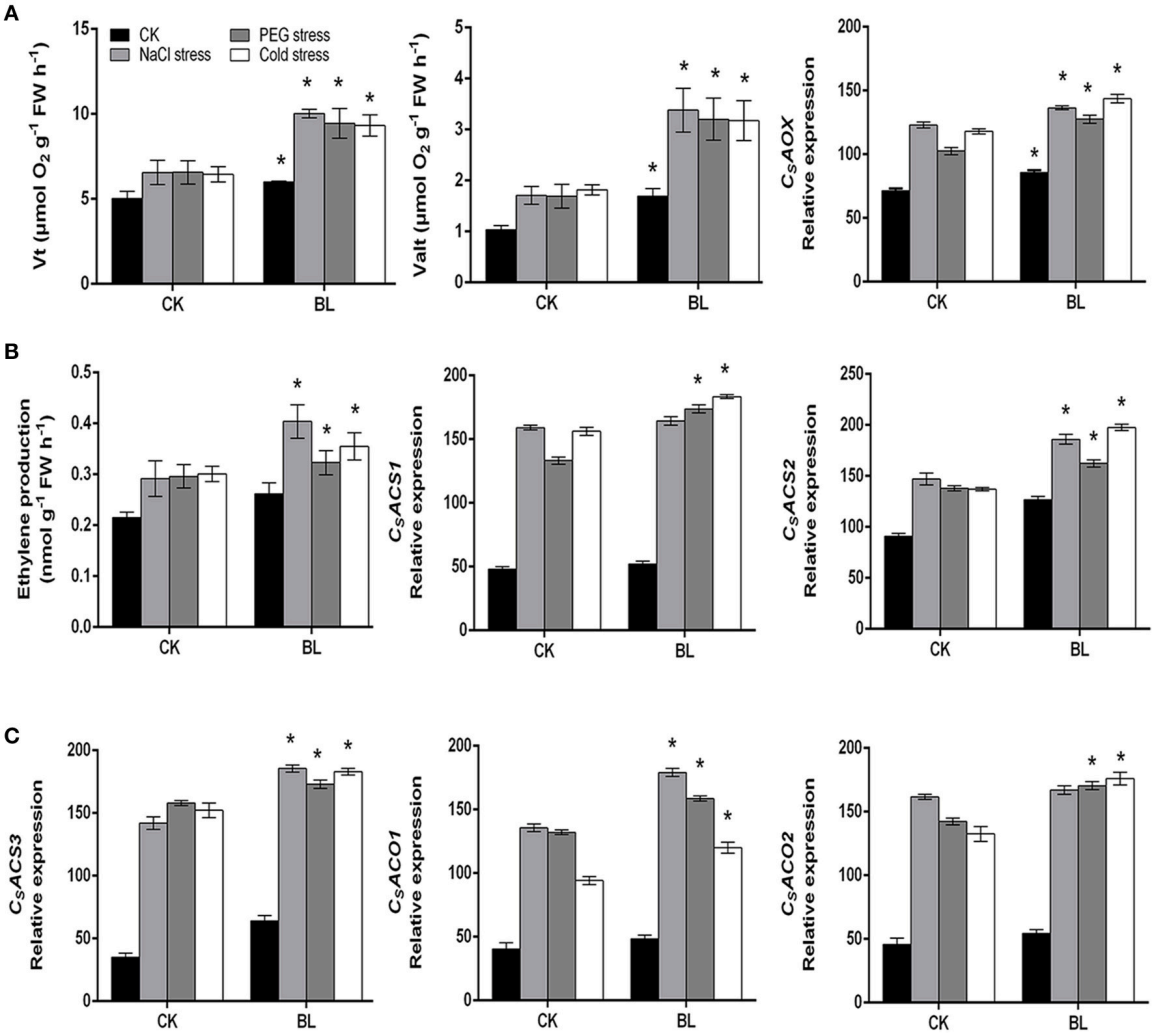
**Changes in respiration rate and *CsAOX* transcript (A), ethylene content and transcripts of *CsACS1*, *CsACS2*, *CsACS3*, *CsACO1*, and *CsACO2* (B,C) in cucumber seedlings under stress conditions for 3 d with or without 1 μM BL pretreatment**. Data are the mean ± SD of three biological repeats; the significant difference was analyzed by Student's *t*-test (^*^P < 0.05) and compared to control (CK).

According to the recent reports (Xia et al., 2009; Jiang et al., 2013; Deng et al., 2015), we know that H_2_O_2_ is induced by BRs and then rapid generation of ROS induces the expression of AOX, which can eliminate ROS to maintain it in safe level. So we investigated whether BRs could further induce the alternative pathway capacity and ethylene biosynthesis under stress conditions in cucumber. Our results showed that both the alternative respiration and total respiration were increased in the BL-pretreated plants as compared to the control after abiotic stresses for 3 d (Figure 2A). Furthermore, under stress conditions, the cyanide-resistant respiration of BL-pretreated seedlings were about 2-fold higher than that of the control. As shown in Figure 2A, consistent with the determined alternative respiration rate, we found that compared with control, BL treatment resulted in an enhanced AOX expression in stress conditions, especially under salt and cold stresses. The content of ethylene was also increased with or without BL pretreatment under stress conditions (Figure 2B). But it worth noting that the production of ethylene was higher in BL-pretreated seedlings than that of the control plants after 3 d of stresses.

We further investigated the transcripts of ethylene biosynthesis genes. Our results suggested that the mRNA levels of *CsACS2, CsACS3*, and *CsACO1* were significantly increased in the BL-pretreated cucumber seedlings in comparison to control plants after 3 d of stresses (Figures 2B,C). However, the transcripts of *CsACS1* and *CsACO2* were higher in the BL treated plants than that of the control seedlings under PEG and cold stresses, respectively. Taken together, these results indicated that both AOX and ethylene might be involved in the BR-induced stress tolerance.

### Ethylene is involved in BR-induced AOX capacity to enhance plant abiotic stresses tolerance

**Figure 3:**
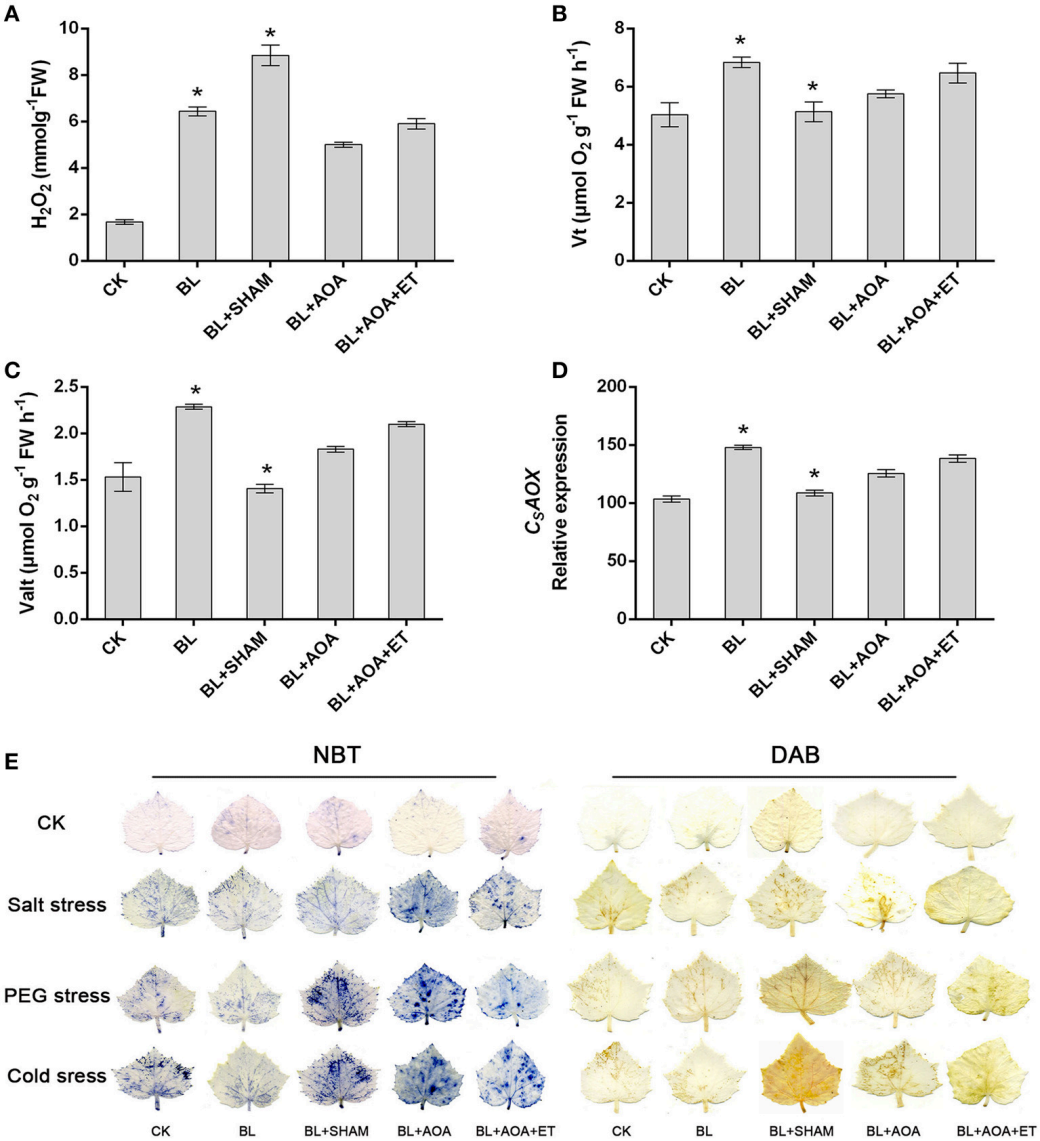
**Effects of SHAM, AOA, or AOA + ET treatment on H_2_O_2_ content (A), respiration rate (B,C), and *CsAOX* mRNA level (D) in the BL-treated cucumber seedlings**. **(E)** NBT and DAB staining were used to detect the presence of superoxide and H_2_O_2_ contents in leaves. Data are the mean ± SD of three biological repeats; the significant difference was analyzed by Student's *t*-test (^*^P < 0.05) and compared to control (CK).

**Figure 4:**
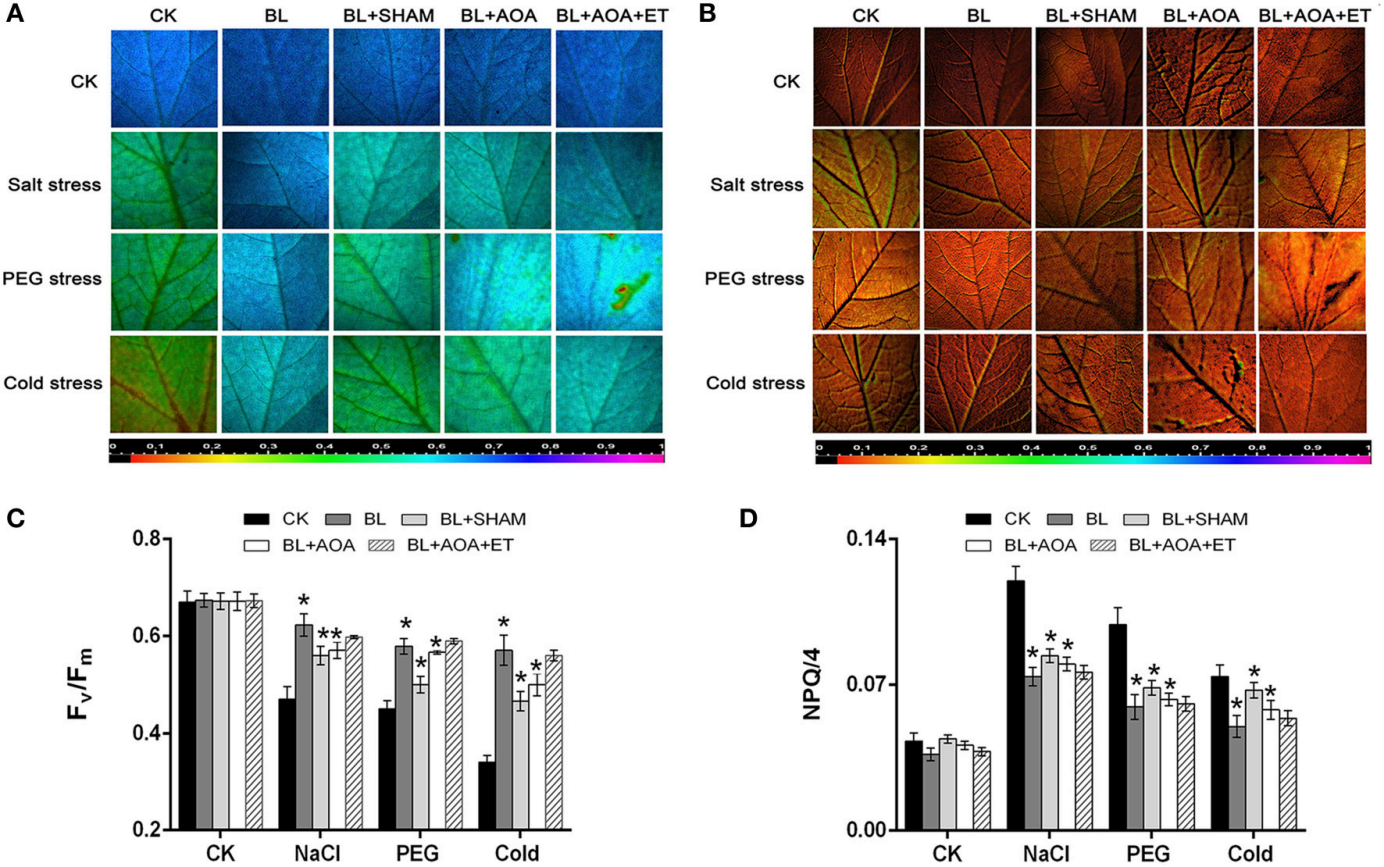
**Effects of AOX or ethylene biosynthesis inhibitors on cucumber seedlings subjected to environmental stress**. Images of the maximum PSII quantum yield (*F*_*v*_/*F*_*m*_) **(A)** and NPQ/4 **(B)** in the leaf of each cucumber seedlings under stress conditions for 3 d. Average values of *F*_*v*_/*F*_*m*_ **(C)** and NPQ/4 **(D)** for the respective chlorophyll fluorescence images. Ten plants were used for each treatment and a picture of one representative leaf is shown. Bars represent mean and standard deviation of values obtained from five biological replicates. The significant difference was analyzed by Student's *t*-test (^*^P < 0.05) and compared to control (CK).

We investigated the change of AOX activity on the plants against different abiotic stress conditions with or without AOA or AOX inhibitor treatment. Our results showed that AOA pretreatment could also inhibit cyanide-resistant respiration and ROS production which were induced by BL treatment. However, the decline was rescued in seedlings pretreated with ET (Figures 3A–E). These results showed that ethylene was involved in BRs-induced AOX activity. Most stress conditions have harmful effects on various biological processes in seedlings, including the photosystem II (PSII). Chlorophyll fluorescence measurements showed that the PSII photochemical efficiency was influenced by stress conditions (Liu et al., 2009). *F*_*v*_/*F*_*m*_ and the non-photochemical quenching (NPQ) are indicators of PSII photochemistry. So, we investigated the function of photosystem II (PSII) under different abiotic stresses treatments. As shown in Figures 4A,C, fluorescence images of *F*_*v*_/*F*_*m*_ showed that compared with control, plants with BL treatment showed an increase in *F*_*v*_/*F*_*m*_ in all three abiotic stresses treatments. In contrast, the non-photochemical quenching (NPQ) declined in the BL-pretreated seedlings in comparison with control plants under the same stress treatments for 3 d (Figures 4B,D). However, BL-induced tolerance to photo-oxidative stress was largely inhibited if the plants were pretreated with AOA or SHAM. Application of ET almost rescued the 1 mM AOA-decreased stress tolerance (Figure 4). These results showed that ethylene is involved in BRs-induced AOX activity and then to enhance plant stress tolerance.

**Figure 5:**
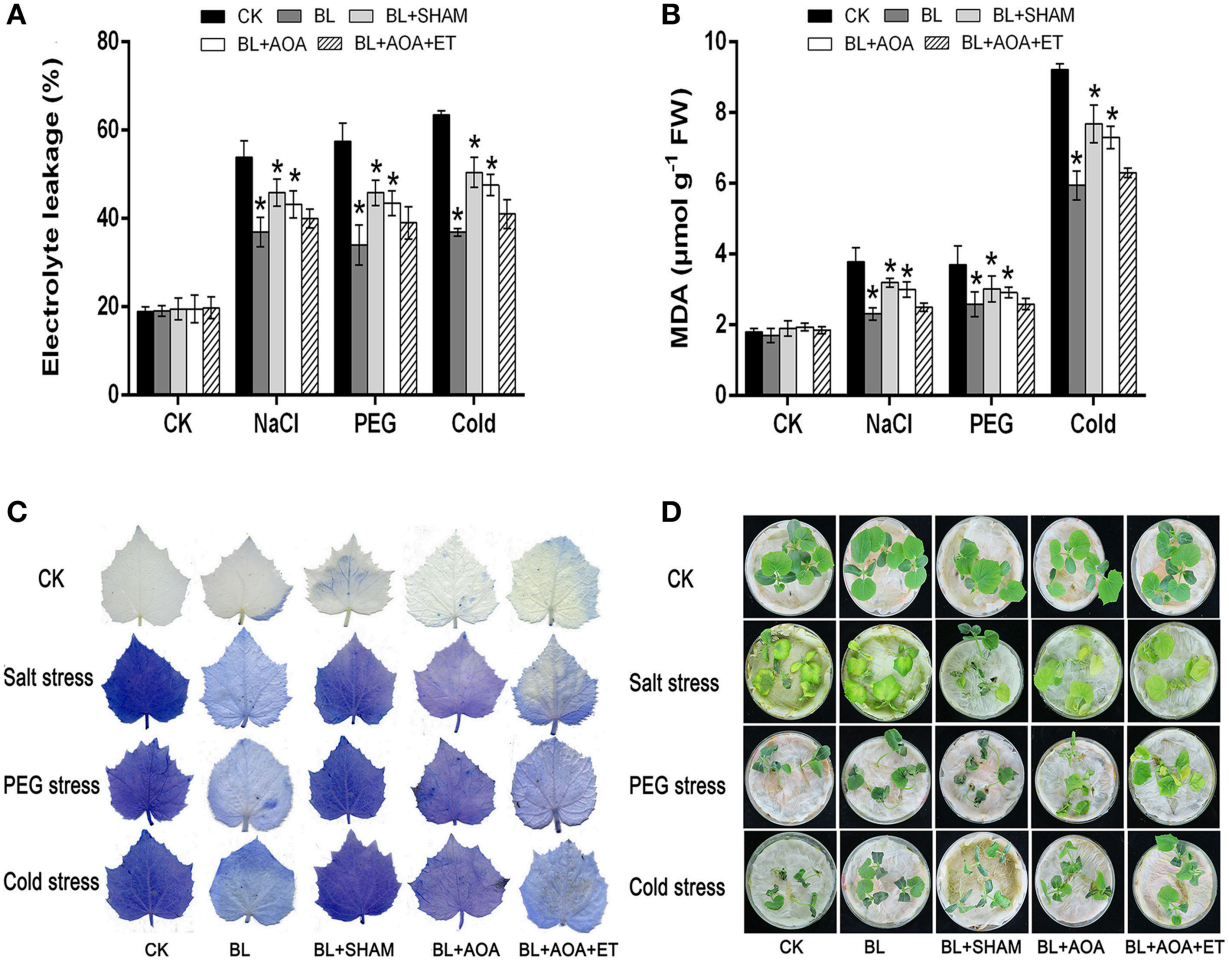
**Effects of AOX or ethylene biosynthesis inhibitors on cucumber seedlings subjected to environmental stress**. Changes in EL **(A)** and MDA content **(B)** under stress conditions for 3 d. **(C)** Cell death was detected by staining with 1.25 mg mL^−1^ of trypan blue. **(D)** Representative phenotypes of cucumber seedlings under stress conditions for 3 d. Data are the mean ± SD of three biological repeats; the significant difference was analyzed by Student's *t*-test (^*^P < 0.05) and compared to control (CK).

We detected the accumulation of H_2_O_2_ and superoxide using DAB and NBT staining procedures, respectively. Other physiological indicators such as EL, MDA content and cell death can also indicate the degree of oxidative damage in seedlings caused by abiotic stresses. The results showed that BL pretreatment can alleviate oxidative damage. However, these protective effects induced by BL were blocked in AOA or SHAM pretreated seedlings (Figures 3E, 5). Moreover, application of ET resumes these protective effects induced by BL in AOA pretreated plants. In a word, our results indicated that BL induced ethylene play a protective role in plant photosystem against stress conditions.

### Ethylene cooperates with H_2_O_2_ to enhance the BL-induced plant abiotic stresses tolerance

**Figure 6:**
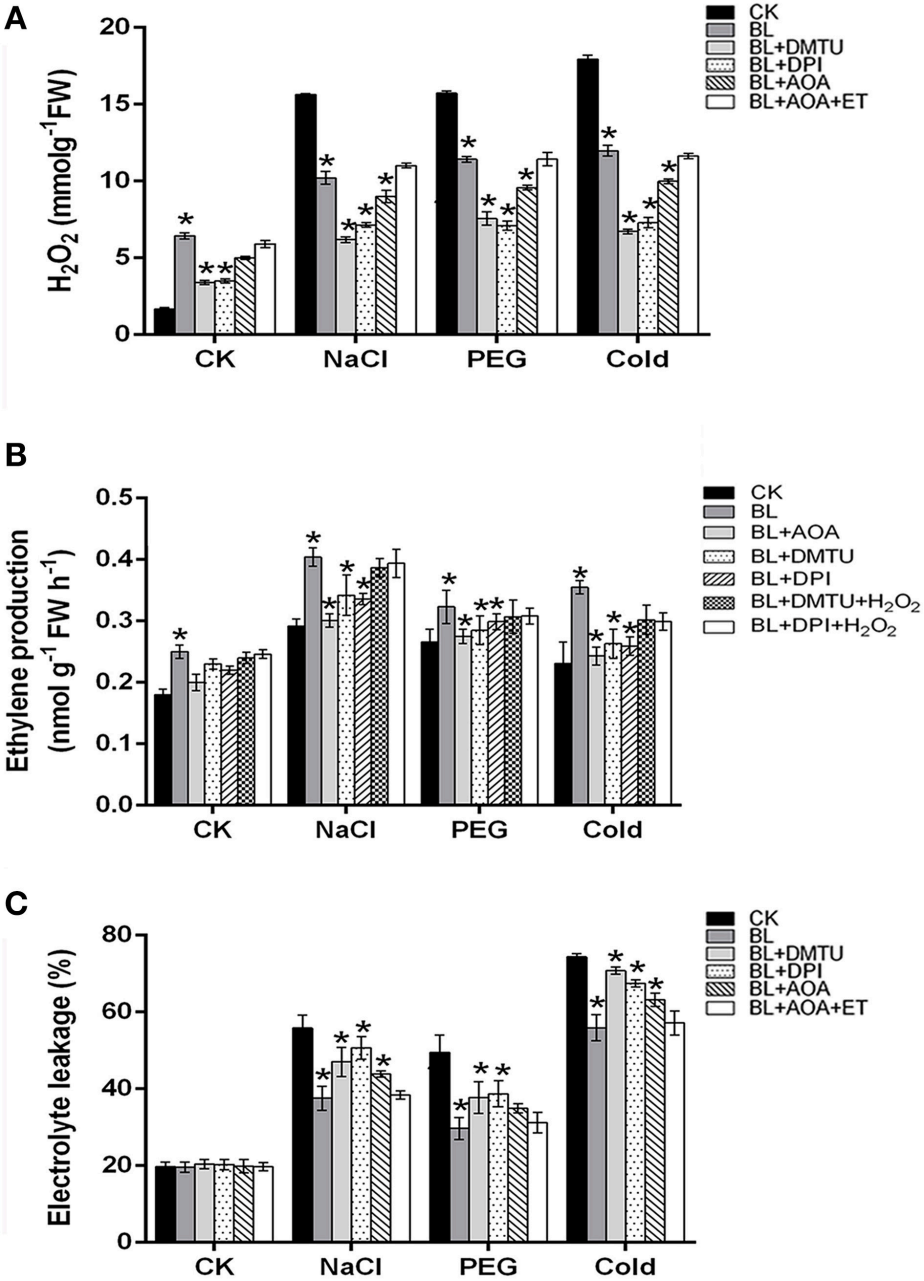
**H_2_O_2_ and ethylene involvement in BR-induced stress tolerance**. **(A–C)** H_2_O_2_, ethylene production and electrolyte leakage (EL) were detected under stress conditions for 3 d. Data are the mean ± SD of three biological repeats; the significant difference was analyzed by Student's *t*-test (^*^P < 0.05) and compared to control (CK).

On the basis of recent reports, we know that H_2_O_2_ and ethylene might function in AOX pathway induction under stresses (Wang et al., 2010b). In order to investigate the relationship of ethylene and H_2_O_2_ in the BL-induced AOX capacity, NADPH oxidase inhibitor DPI, H_2_O_2_ scavenger DMTU, ethylene biosynthesis inhibitor AOA, H_2_O_2_, and ethylene were used in this study. As shown in Figure 6, application of 5 mM DMTU, 100 μM DPI or 1 mM AOA to cucumber seedlings had significantly down-regulated on H_2_O_2_ content and ethylene generation, while EL was increased compared with that of the BL treatment plants. However, the decline of H_2_O_2_ content and ethylene generation were rescued in seedlings pretreated with ET or H_2_O_2_. These results demonstrated that ethylene cooperates with H_2_O_2_ played important roles in the BR-induced abiotic stresses tolerance.

### Ethylene and H_2_O_2_ are located in the upstream of BR-induced AOX pathway to enhance plant abiotic stresses tolerance

**Figure 7:**
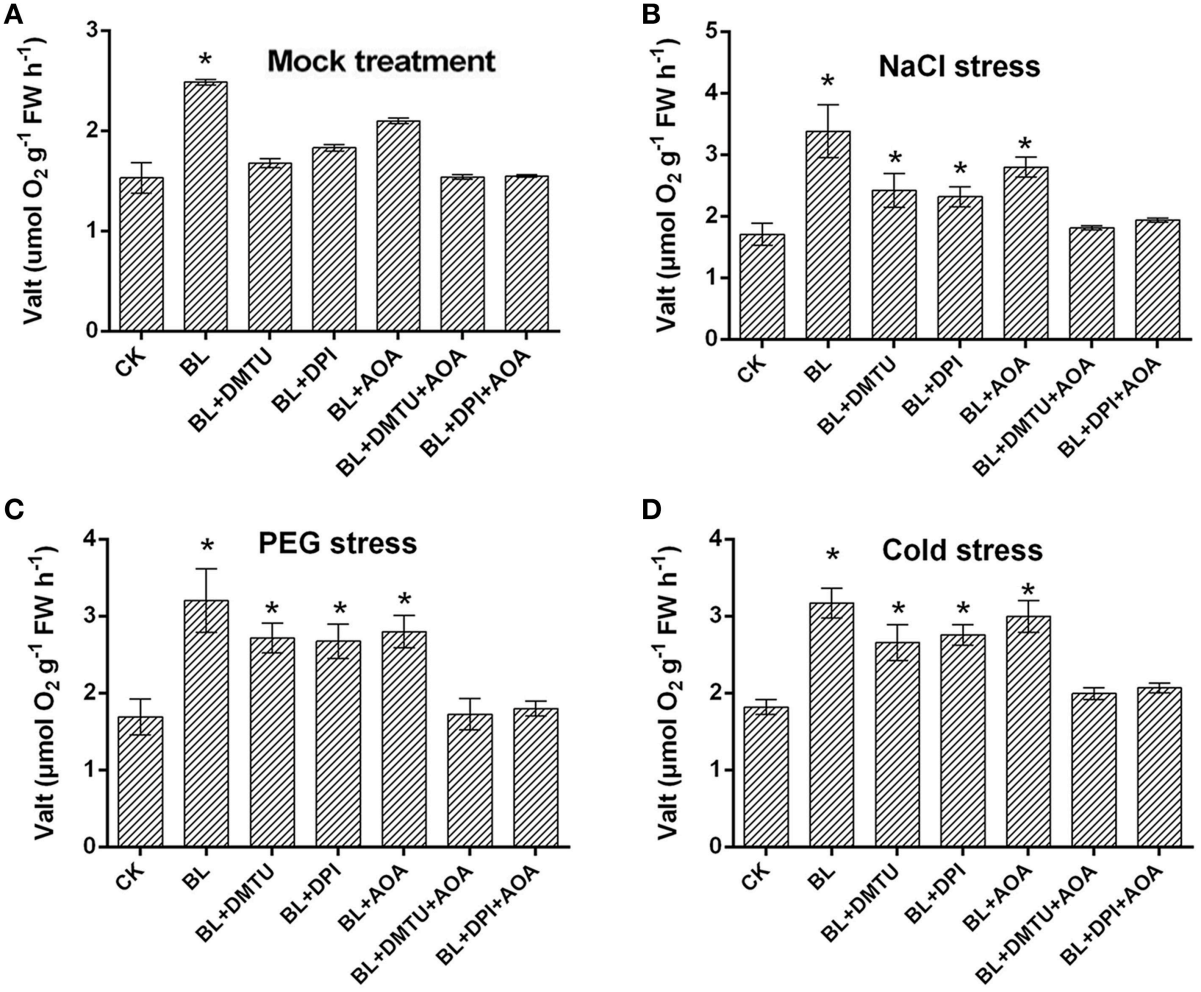
**Effects of H_2_O_2_ and ethylene inhibitors on BRs-induced AOX capacity in response to environmental stress**. **(A–D)** Plants were pretreated with 100 μM DPI, 5 mM DMTU for 8 h or 1 mM AOA for 12 h, then plants were treated with 1 μM BL 12 h later, plants were challenged with stress conditions for 3 d. Data are the mean ± SD of three biological repeats; the significant difference was analyzed by Student's *t*-test (^*^P < 0.05) and compared to control (CK).

To examine whether there were other factors involved in the BR-induced AOX pathway, the respiration rate was investigated in the cucumber seedlings treated with BL, DMTU/DPI, AOA, and DMTU/DPI+ AOA. As shown in Figure 7, compared with BL-pretreated plants, BL induced cyanide-resistant respiration was decreased in BL+DMTU/DPI and BL+AOA treated plants under environment stresses. Besides, the cyanide-resistant respiration rate of the plants treated with BL+ DMTU/DPI+AOA was strongly inhibited but still increased slightly as compared with the control. These results indicated that ethylene and hydrogen peroxide were involved in the BR-induced AOX pathway, as well as other factors.

### BL alleviates oxidative damage and activates antioxidant system in cucumber

H_2_O_2_ is a vital type of ROS in seedling cells, other indicators such as EL and MDA content can also show the degree of oxidative damage in seedlings caused by stress conditions. Consistent with the observed damage of cucumber plants in Figure 5D, the BL-pretreated cucumber showed lighter stress damage than that of the control. To demonstrate these results, H_2_O_2_ level and superoxide content were detected by DAB and NBT staining (Figure 3E), which were consistent with the phenotypes of seedlings. Meanwhile, under the same stress condition, the BL-pretreated plants maintained lower MDA and EL than the control (Figures 5A,B).

**Figure 8:**
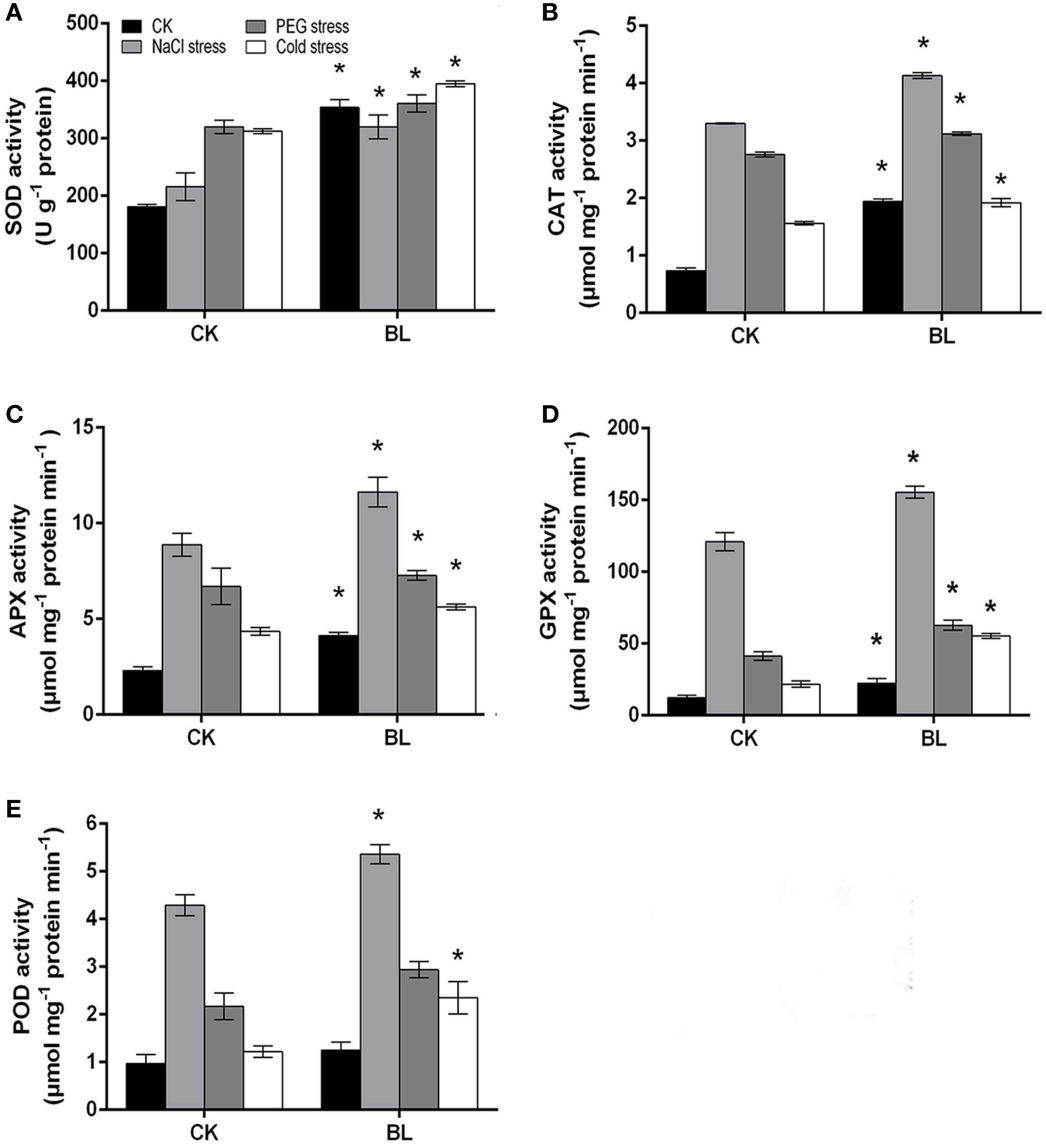
**Antioxidant enzymes activities in water and 1 μM BL pretreated cucumber seedlings under stress conditions for 3 d**. Leaf samples were harvested at 12 h with BL pretreatment and the activities of antioxidant enzymes were analyzed. SOD **(A)**, CAT **(B)**, APX **(C)**, GPX **(D)**, POD **(E)**. Data are the mean ± SD of three biological repeats; the significant difference was analyzed by Student's *t*-test (^*^P < 0.05) and compared to control (CK).

According to the recent research, the antioxidant defense machinery protects plants against oxidative stress damages (Foyer and Noctor, 2009; Xu et al., 2012a). As shown in Figure 8, all antioxidant enzyme activities increased in the presence or absence of BL under stress treatments. However, the activities of APX, GPX, and CAT were pronouncedly increased with BL pretreatment, and their levels were approximately 1.3-fold higher compared with the control seedlings after 3 d of stresses. Besides, the activity of POD in BL treated plants showed a similar trend to the control. In addition, we determined the mRNA levels of antioxidant enzyme genes. The results indicated the transcripts of *C*_*S*_*POD24, C*_*S*_*SOD1*, and *C*_*S*_*SOD2* were much higher in the BL-treated cucumber seedlings than in the non-treated after 3 d of stresses (Supplemental Figure S2). It has been showed that the antioxidative system was involved in polyunsaturated fatty acid (PUFA) metabolism (Lee et al., 2005; Cho et al., 2012). Whether, BR could regulate PUFA metabolism will be investigated in the future.

## Discussion

Under salt, PEG and cold-stresses, cucumber seedlings displayed enhanced CN-resistant respiration and ethylene content after BL treatment. In addition, transcriptional levels of the AOX and ethylene biosynthesis related genes were also induced in the BL-pretreated seedlings as compared to the control (Figure 2). These results suggested that BRs could enhance ethylene content and AOX capacity. Our results confirmed the previous reports that the enhanced AOX and ethylene contribute to plant abiotic stresses tolerance (Kendrick and Chang, 2008; Xu et al., 2012b). It was reported that ethylene biosynthesis pathway was connected with AOX capacity in tomato (Xu et al., 2012a). When ethylene level is low under normal condition, it is not sufficient in itself to induce AOX activity (Wang et al., 2010b). On the contrary, when ethylene content maintains at high level in seedlings pretreated with BL under stress conditions, the activated ethylene signal by BRs can activate downstream targets which induce AOX activity (Figures 2, 7). In this study, CN-resistant respiration was largely impaired by AOA treatment (Figure 7). Thus, our results, together with previous research, suggest that ethylene is involved in BRs-induced AOX capacity.

In the present work, BL-treated plants showed a significant increase in *F*_*v*_/*F*_*m*_ and a decline in NPQ under abitotic stress conditions for 3 d (Figure 4). However, these BRs-induced protective effects, expressed as *F*_*v*_/*F*_*m*_ and NPQ were mostly inhibited if the seedlings were pre-treated with SHAM or AOA (Figure 4). Addition of ET rescued the decreased stress tolerance tendency by AOA treatment (Figures 3E, 4, 5). Our study demonstrates that ethylene is involved in BRs-induced AOX capacity. Under abiotic stress conditions, the inhibition of AOX and ethylene resulted in more dissipated excitation energy and lower level of photochemical efficiency (Figure 4), similar to the previous study (Giraud et al., 2008). Previous studies showed that the mitochondrial AOX pathway might play a pivotal role in the protection of plants photosystem by alleviating the inhibition of the repair of the photodamaged PSII (Zhang et al., 2011; Deng et al., 2015), and inhibition of the alternative pathway leaded to decreases in photosynthetic rate in seedlings (Yoshida et al., 2006; Zhang et al., 2010). Thus, these findings, together with previous research, suggest that the enhancement of AOX and ethylene by BRs might contribute to balancing chloroplast-to-mitochondria electron transfer, and thus decrease ROS accumulation in cucumber seedlings.

**Figure 9:**
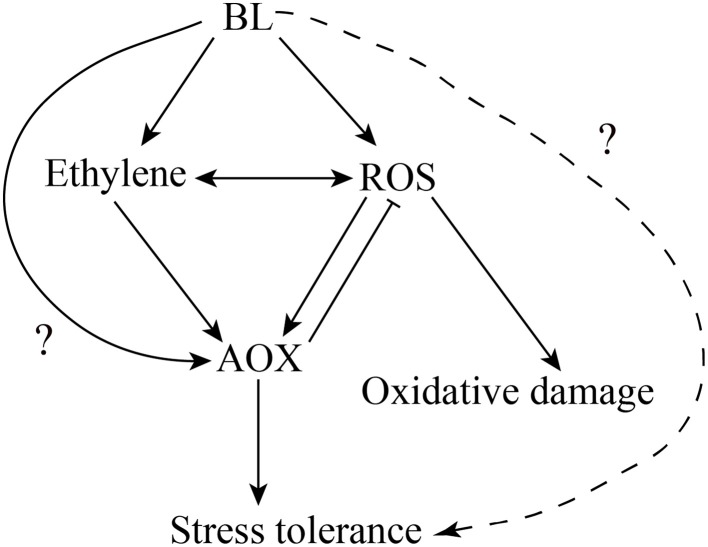
**Model illustrating the hypothetical function of BL, ethylene and ROS in AOX induction in cucumber seedlings under stress**.

Xia et al. (2011) have proved that H_2_O_2_ play an important role in BRs-induced stress tolerance. Firstly, BR-induced stress tolerance was promoted by increased H_2_O_2_ level. Secondly, either scavenging of H_2_O_2_ or inhibiting H_2_O_2_ generation abolished BR-induced stress tolerance. H_2_O_2_, as a signaling molecule, is involved in multiple plant response to environmental stresses (Neill et al., 2003; Mittler et al., 2004; Zhang et al., 2007; Wang et al., 2010b). Feng et al. (2008) and Wang et al. (2010a) also confirmed H_2_O_2_ could induce AP and AOX1 expression under chilling and salinity conditions. Recently, we studied whether H_2_O_2_ is involved in the BRs-induced AOX capacity (Deng et al., 2015). Here, we confirmed that H_2_O_2_ may play a vital role in BR-induced AOX activity in cucumber. To demonstrate this assumption, the respiration rate was investigated in the cucumber seedlings pretreated with 5 mM DMTU or 100 μM DPI. Moreover, compared with BL-treated plants, cyanide-resistant respiration was weaker in BL+ DMTU/DPI pretreated plants under environment stresses. Thus, it indicated that H_2_O_2_ played a role in BR-induced AOX activity in cucumber (Figures 6, 7). Further, investigations were conducted to make clear the relationship between H_2_O_2_ and ethylene in AOX induction under abiotic stress conditions. The results showed that ethylene cooperated with H_2_O_2_ to induce AOX capacity under stresses (Figure 6). According to the results presented here, also those reported previously (Ederli et al., 2006; Giraud et al., 2008; Wang et al., 2009, 2010b; Xia et al., 2009), we present a hypothetical pattern in cucumber seedlings describing the interrelationships among BL, ethylene, ROS, the AOX and abiotic stress tolerance. In this pattern, BL induces H_2_O_2_ and ethylene generation, which subsequently enhances AOX capacity. The enhanced AOX activity can eliminate superfluous H_2_O_2_ generation to avoid oxidative damage in plant cells, and then improve stress tolerance. Besides, we don't know if there are other factors such as transcription factors and kinase in BR signaling pathway involved in AOX regulation in addition to ethylene and H_2_O_2_ (Figure 9). BRs may also regulate plant stress tolerance through affecting other mechanisms. Exogenous application of BRs improved the Cu and Cr stress tolerance by inducing endogenous ABA, auxin, and polyamine profiles in radish (*Raphanus sativus*), which suggested the potential crosstalk between BRs and ABA or auxin in stress responses (Choudhary et al., 2012). Furthermore, BRs may affect other pathways to activate antioxidative enzyme (Choudhary et al., 2010). Epibrassinolide induces the change in indole-3-acetic acid, abscisic acid and polyamine concentrations and enhances antioxidant potential of radish seedlings under copper stress (Choudhary et al., 2011). Epibrassinolide ameliorates Cr stress tolerance via influencing the levels of indole-3-acetic acid, abscisic acid, polyamines and antioxidant system of radish seedlings.

In brief, suitable concentrations of BL positively affected the tolerance of plant to stress conditions. AOX may play an important role in the plant resistance to environment stresses. Our study showed that both ethylene and H_2_O_2_ were involved in BRs-induced AOX activity. Thus, our results reveal a novel role of BRs in plant against environmental stresses and clarify the relationships between ethylene, AOX and ROS during environmental stress, although the detailed mechanism relations need further investigation. Future, investigations should focus on whether BRs could directly induced AOX activity by BR signaling.

### Conflict of interest statement

The authors declare that the research was conducted in the absence of any commercial or financial relationships that could be construed as a potential conflict of interest.

## Supplementary material

Supplemental Figure S1 **(A)** Representative phenotypes of cucumber seedlings under stresses for 3 days. Cucumber were pretreated with 0.1, 0.5, 1, 5, or 10 μM BL while the control plants were pretreated with distilled water for 12 h, respectively, and then all the seedlings were subjected to salt (200 mM NaCl), PEG (16% PEG 6000) and cold stresses (at 4°C) for 3 days. **(B)** Change in the content of H_2_O_2_ in the leaves of cucumber plants, plants were pretreated with 1 μM BL for 12 h, then plants subjected to stresses.

Supplemental Figure S2 **Changes in the expression of antioxidant enzyme related genes in cucumber seedlings under stress conditions for 3 days**. Transcripts of *CsSOD1, CsSOD2, CsPOD2, CsPOD24, CsCAT*, and *CsAPX* were determined by qRT-PCR. Data are the mean ± SD of three biological repeats; the significant difference was analyzed by Student's *t*-test (^*^*P* < 0.05) and compared to control (CK).

Supplemental Table S1 **Sequences of primers used in real time qRT-PCR experiments**.
