# Supplementary figures and images for: Ethylene is Involved in Brassinosteroids Induced Alternative Respiratory Pathway in Cucumber (Cucumis sativus L.) Seedlings Response to Abiotic Stress
Source: Front Plant Sci. 2015 Nov 10;6:982. doi: 10.3389/fpls.2015.00982 (PMC4639706; doi:10.3389/fpls.2015.00982)

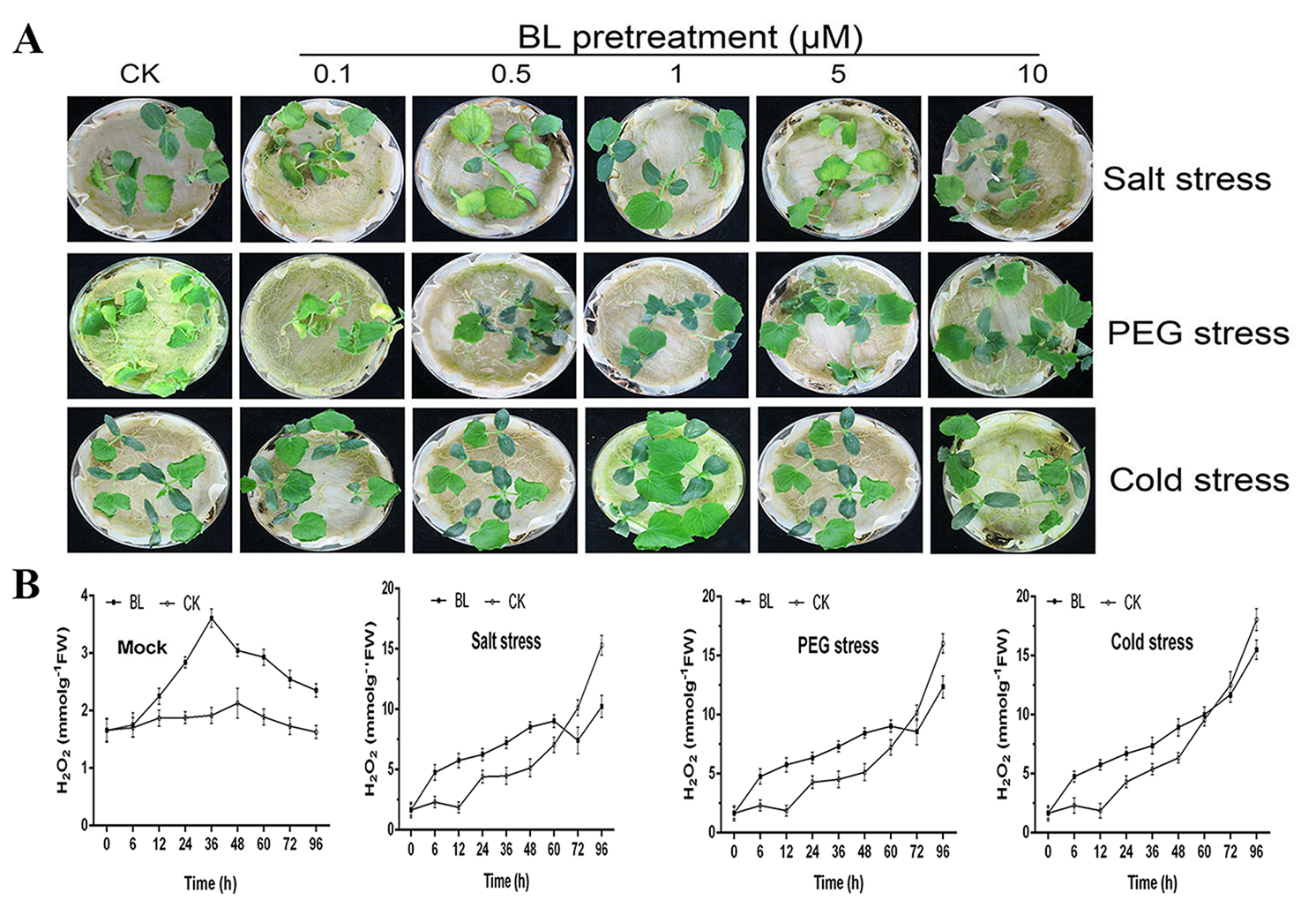

Supplement: Supplemental Figure S1 — (A) Representative phenotypes of cucumber seedlings under stresses for 3 days. Cucumber were pretreated with 0.1, 0.5, 1, 5, or 10 μM BL while the control plants were pretreated with distilled water for 12 h, respectively, and then all the seedlings were subjected to salt (200 mM NaCl), PEG (16% PEG 6000) and cold stresses (at 4°C) for 3 days. (B) Change in the content of H2O2 in the leaves of cucumber plants, plants were pretreated with 1 μM BL for 12 h, then plants subjected to stresses. [file Image1.TIF]

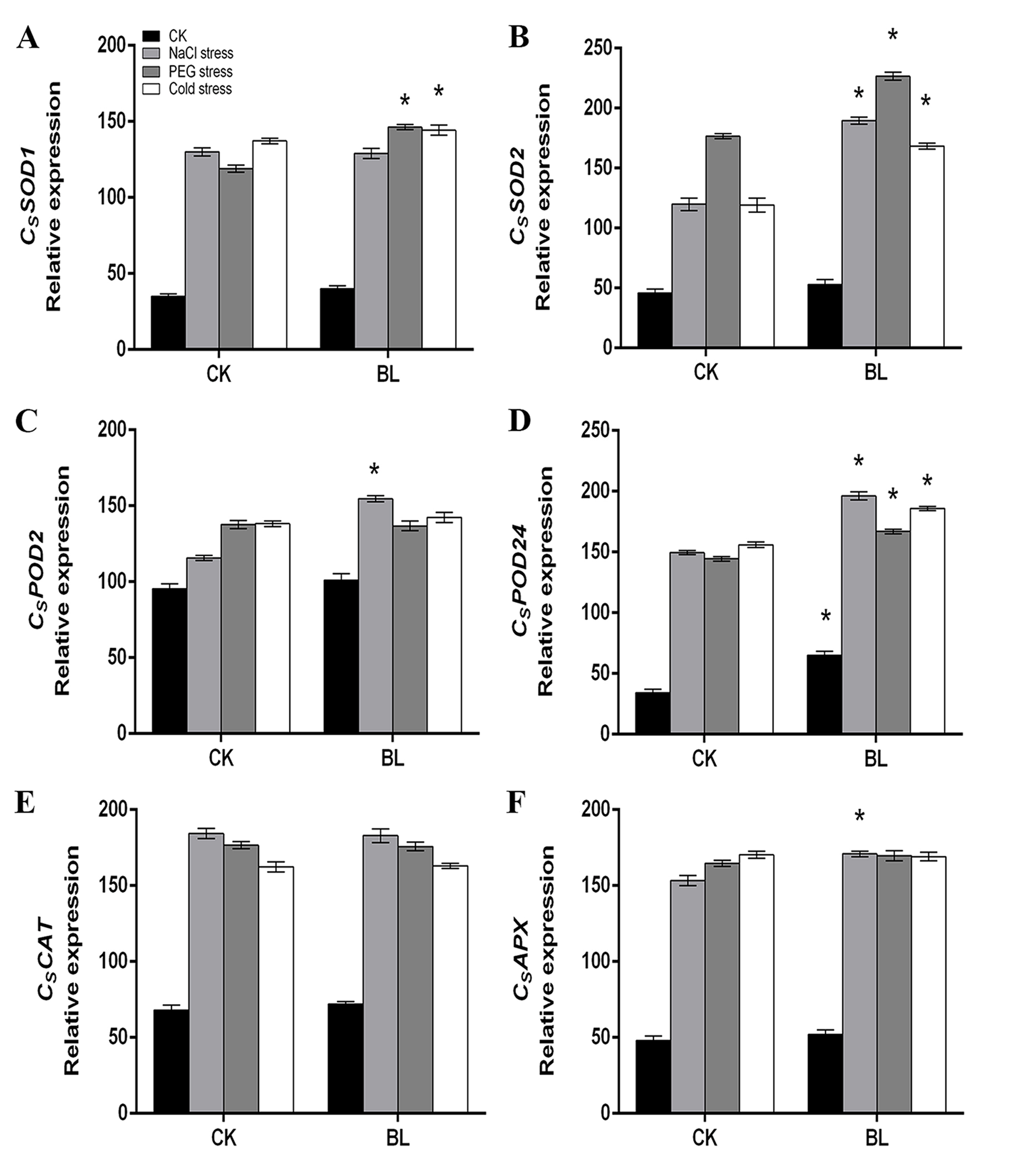

Supplement: Supplemental Figure S2 — Changes in the expression of antioxidant enzyme related genes in cucumber seedlings under stress conditions for 3 days. Transcripts of CsSOD1, CsSOD2, CsPOD2, CsPOD24, CsCAT, and CsAPX were determined by qRT-PCR. Data are the mean ± SD of three biological repeats; the significant difference was analyzed by Student's t-test (*P < 0.05) and compared to control (CK). [file Image2.TIF]
