# Supplementary material for: Ethylene is Involved in Brassinosteroids Induced Alternative Respiratory Pathway in Cucumber (Cucumis sativus L.) Seedlings Response to Abiotic Stress
Source: Front Plant Sci. 2015 Nov 10;6:982. doi: 10.3389/fpls.2015.00982 (PMC4639706; doi:10.3389/fpls.2015.00982)
Supplement: Supplemental Table S1 — Sequences of primers used in real time qRT-PCR experiments. [file Table1.DOCX]

**SUPPORTING INFORMATION**

Supplemental Table S1 Sequences of primers used in real time qRT-PCR experiments.

| Gene | Accession number | Forward primer | Reverse primer |
| --- | --- | --- | --- |
| *CsAOX* | *DQ641114* | TCATCATCACCGAACTTACA | GAATCCACCATCCGACAA |
| *CsACS1* | *U59813.1* | CTTCAGGCGTTATTCAGATG | GTGCTCGTGTTCTCCATT |
| *CsACS2* | *AB006804* | GGTCTACTCATAACGAATCCT | TGCTTCCAATGTCTTCTCTT |
| *CsACS3* | *AB006805* | CCAACGGCATCATTCAGA | GCAAGGCAGAACATAAGTG |
| *CsACO1* | *AB006806* | ACCTTCTTCTTACGCCATC | GCCACCTACCTTGTCATC |
| *CsACO2* | *AB006807* | GAACCACGGAATAGATACAGA | GGAGATGACGGAGGAAGA |
| *CsSOD1*  *CsSOD2*  *CsPOD2*  *CsPOD24*  *CsCAT*  *CsAPX*  *ACTIN* | *NC026658 NC026655*  *M91372*  *XM004153791*  *XP011654421*  *AY338957*  *DQ641117* | GAGCAATCAGGGAGTCAGT  ATGGGAATGGATACGAGAC  CTGCCAATAGCACAAGAAG  TTCTTGCCCTTCAGGTTGT  AACAACACCGCCGTAATGT  ATGGCACTCTGCTGGAAC  AGAGATGGCTGGAATAGAAC | TACCATCATCACCAGCAAC CTGCCAATAGCACAAGAAG GCCTATCCCTGGCTCCTTG CTCCGATTGATTTGTTCCA ATGACGGGGGTTTGGACG GTCTGCATATGAGAGGATGG  CTGGTGATGGTGTGAGTC |
